# Aorto-Right Ventricular Tunnel in Transposition of the Great Arteries

**DOI:** 10.3389/fped.2018.00030

**Published:** 2018-02-21

**Authors:** Antonio F. Corno, Saravanan Durairaj, Robert H. Anderson

**Affiliations:** ^1^Cardiovascular Research Center, University of Leicester, Leicester, United Kingdom; ^2^East Midlands Congenital Heart Center, University Hospitals of Leicester, Glenfield Hospital, Leicester, United Kingdom; ^3^Institute of Genetic Medicine, International Centre for Life, Newcastle University, Newcastle upon Tyne, United Kingdom

**Keywords:** annular hinge, aorto-ventricular tunnel, arterial switch, new pulmonary valve regurgitation, semilunar valves

## Abstract

Aorto-ventricular tunnel is an extremely rare congenital heart defect, consisting of failure of attachment of an aortic leaflet along the semilunar hinge. In all published reports the leaflet involved was either the right coronary leaflet, most frequently, or the left coronary leaflet, in most of the cases opening toward the left ventricle, with only one-eighth of the reported cases communicating with the right ventricle. Treatment of the aorto-ventricular tunnel has been anecdotally reported by interventional closure with a device and more frequently with surgical approach, either as an isolated malformation or as associated lesions. To the best of our knowledge, the presence of an aorto-ventricular tunnel of the non-adjacent aortic leaflet in transposition of the great arteries has never been reported. We have observed an aorto-ventricular tunnel involving the non-adjacent leaflet of the aortic root, which after arterial switch became the pulmonary root. The patient presented 18 years after the arterial switch with progressive dilatation of the right ventricle due to severe degree of pulmonary valve regurgitation, confirmed by echocardiography and cardiac MRI. Indication for surgery was given with the plan for a pulmonary valve implantation. Because of the intra-operative finding of disconnection of the anterior leaflet of the pulmonary valve (former aortic valve) along the semilunar hinge, the surgical plan was modified and the anterior leaflet was attached to the valve annulus, with subsequent plasty in correspondence with the right and left commissurae to reduce the size of the dilated annulus to normal diameter. The post-operative course was uneventful, with extubation after few hours and discharge 4 days after surgery, with echocardiography showing trivial degree of pulmonary valve regurgitation. The patient remains in good conditions 6 months after surgery.

## Introduction

Aorto-ventricular tunnel is an extremely rare congenital heart defect (1–25), consisting of failure of attachment of an aortic leaflet along the semilunar hinge (24, 25). In all published reports, the leaflet involved was either the right coronary leaflet, most frequently, or the left coronary leaflet (1–25), in most of the cases opening toward the left ventricle, with only one-eighth of the reported cases communicating with the right ventricle (24, 25). Treatment of the aorto-ventricular tunnel has been anecdotally reported by interventional closure with a device (26) and more frequently with surgical approach, either as an isolated malformation (27–62) or with associated lesions (63–70).

To the best of our knowledge, the presence of an aorto-ventricular tunnel of the non-adjacent aortic leaflet in transposition of the great arteries has never been reported. We have observed an aorto-ventricular tunnel involving the non-adjacent leaflet of the aortic root, which after arterial switch became the pulmonary root.

## Background

Because of severe cyanosis in the presence of transposition of the great arteries and ventricular septal defect, a neonate underwent emergency balloon atrio-septostomy procedure at the age of 2 weeks. After few days, arterial switch and closure of ventricular septal defect were performed with uneventful post-operative course. The child was then followed with regular follow-up visits; at the age of 3 years, a follow-up cardiac catheterization showed normal reimplanted coronary arteries. From the age of 1 year, the echocardiography reports noted the presence of moderate degree of pulmonary valve regurgitation, increasing to severe degree around the age of 9 years, with the patient remaining asymptomatic. Since after, the echocardiographic investigations showed a progressive dilatation of the right ventricle, confirmed by MRI at the age of 17 years, showing an indexed end-diastolic right ventricular volume = 211 ml/m^2^.

At the 18 years of age, 64 kg of body weight, he presented with mild right ventricular impulse on the chest on examination, and systo-diastolic murmur compatible with free pulmonary valve regurgitation. Electrocardiography showed sinus rhythm with 53’ heart rate, partial right bundle branch block with QRS duration = 110 ms. Echocardiography showed severe pulmonary valve regurgitation, with Doppler velocity = 2.7 m/s through the pulmonary valve.

**Figure 1:**
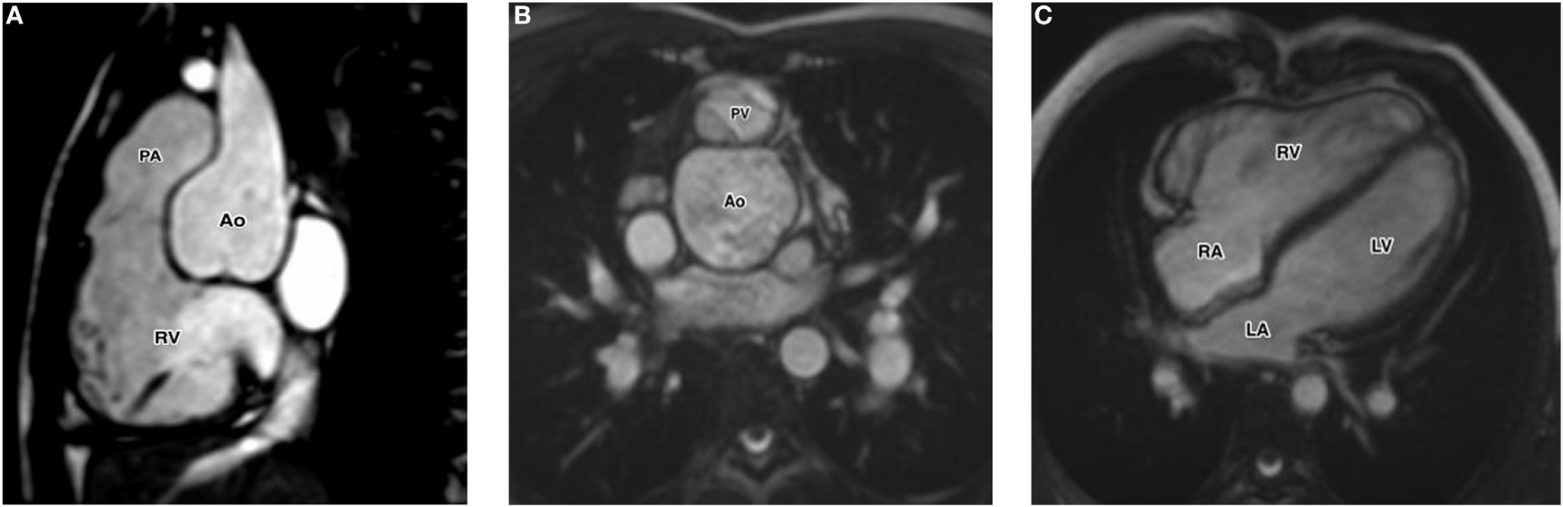
**(A)** Cardiac MRI with sagittal view of the right ventricular outflow tract showing the anterior leaflet of the pulmonary valve without attachment to the annular hinge. **(B)** Cardiac MRI with axial view of the pulmonary valve showing morphologic abnormality of the anterior leaflet. **(C)** Cardiac MRI with four-chamber views showing the dilatation of the right ventricle. Abbreviation: LA, left atrium; LV, left ventricle; RA, right atrium; RV, right ventricle.

Cardiac MRI confirmed the presence of severe pulmonary valve regurgitation with fraction of regurgitation = 57%, dilated pulmonary valve annulus (23 mm, with 18.5 mm normal for the body surface area) (Figures 1A,B), severe dilatation of the right ventricle (Figure 1C), with ejection fraction = 50%, indexed end-diastolic right ventricular volume = 226 ml/m^2^, indexed end-systolic right ventricular volume = 113 ml/m^2^, normal size and function of the left ventricle (Figure 1C). CT scan confirmed the findings, showing mildly dilated ascending neo-aorta and normal coronary arteries.

Cardiopulmonary stress test showed peak VO2 = 39 ml/kg/min (91% of normal), and peak heart rate 157’ (78% of normal). The test was terminated before reaching the maximum heart rate because of ST depression in V3 and V4, without chest pain.

At the multi-disciplinary meeting, the decision was agreed to proceed with pulmonary valve implantation, because of the severe degree of pulmonary valve regurgitation and right ventricular dilatation. The patient was informed and consent was given for the planned surgical procedure.

**Figure 2:**
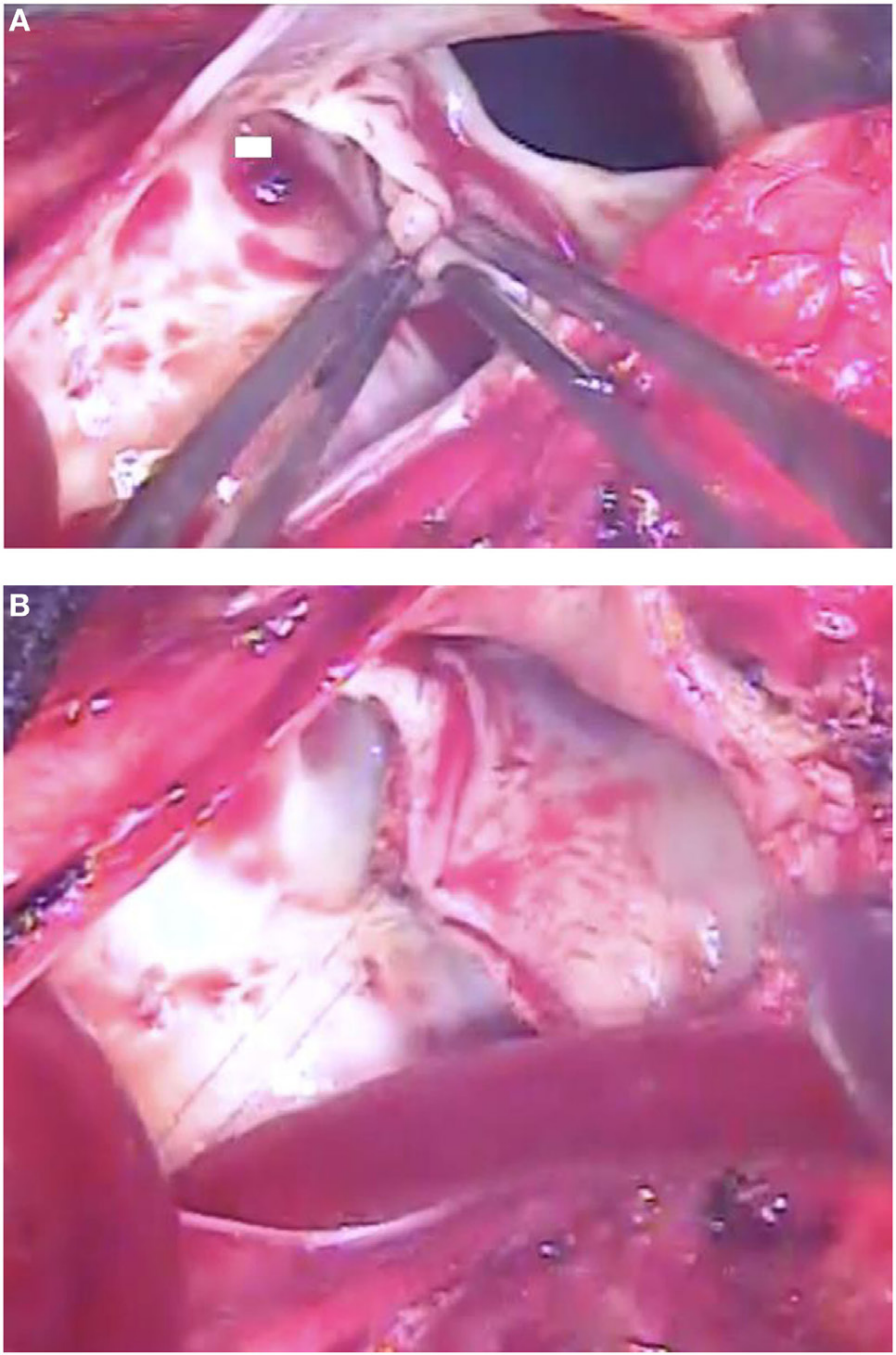
**(A)** Intra-operative photograph showing the anterior leaflet of the pulmonary valve without any attachment to the annular hinge, while the other two leaflets are controlled with forceps. **(B)** Intra-operative photograph showing the anterior leaflet of the pulmonary valve reattached to the annulus with running suture.

On general anesthesia, transesophageal echocardiography confirmed the presence of severe regurgitation of the pulmonary valve. The cardiopulmonary bypass, established between the left femoral artery and the right atrium, was maintained through the entire procedure on normothermia with beating heart. Through a longitudinal incision of the main pulmonary artery, in anterior position after the previous Lecompte maneuver, the presence of three leaflets pulmonary valve (native aortic valve) was confirmed, but while the two posterior leaflets appeared absolutely normal, the anterior non-adjacent leaflet was completely disconnected from the annulus and floating inside the pulmonary artery (Figure 2A). The disconnected anterior non-adjacent leaflet was reattached to the annulus with double running suture of 5/0 monofilament (Figure 2B). At this point, the pulmonary valve annulus, dilated to 24 mm diameter, was reduced to 19 mm diameter (normal for the age = 18.5 mm) with plasty in correspondence of the right and left commissurae with 5/0 monofilament suture pledgeted with a small patch of autologous pericardium. After closure of the incision of the main pulmonary artery with double running suture of 5/0 monofilament, the patient was easily weaned from cardiopulmonary bypass after 35 min, without inotropic support.

**Figure 3:**
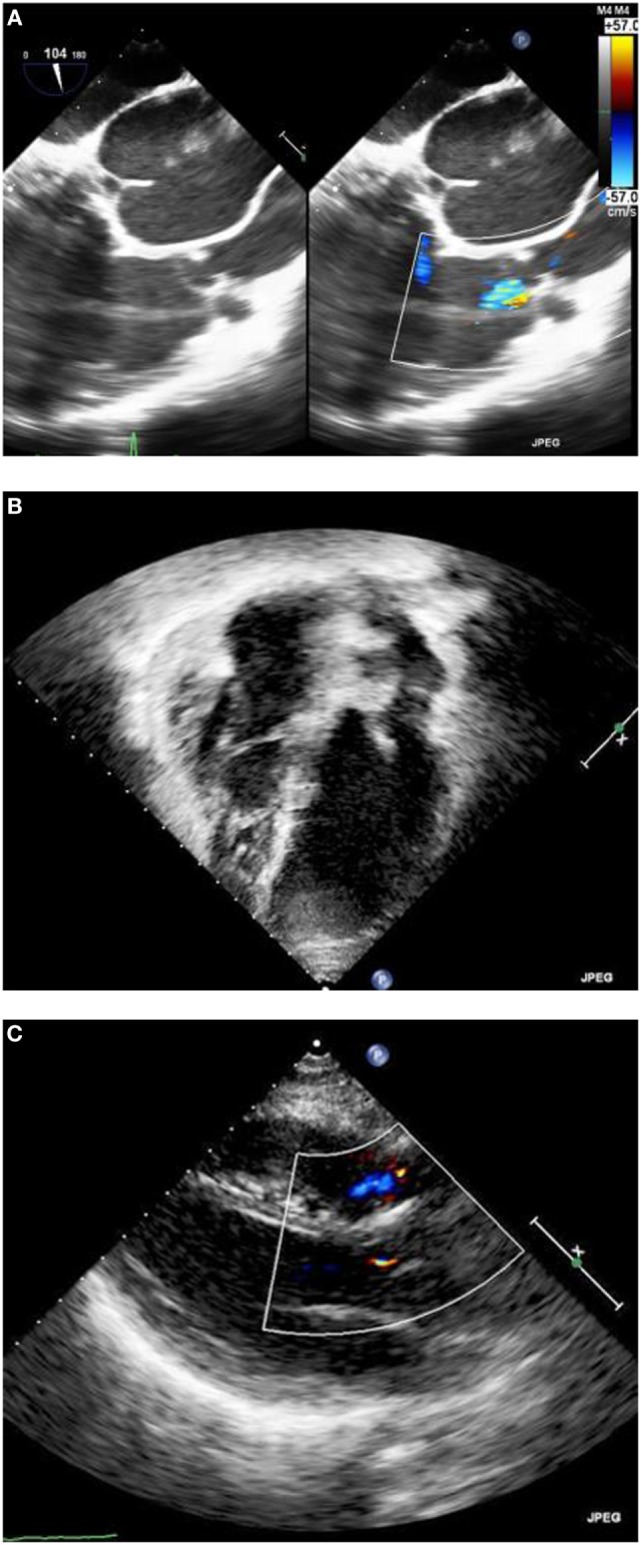
**(A)** Intra-operative transesophageal echocardiography after repair, showing trivial residual degree of pulmonary valve regurgitation. **(B)** Pre-discharge trans-thoracic echocardiography with the four chamber views. **(C)** Pre-discharge trans-thoracic echocardiography with the long-axis view.

Intra-operative transesophageal echocardiography confirmed the good result with trivial regurgitation of the pulmonary valve (Figure 3A). The rest of the hospitalization was uneventful, with extubation after few hours, discharge from ICU next morning and discharge home on the fourth post-operative day. Echocardiography before discharge confirmed the presence of well functioning pulmonary valve and reduced the size of the right ventricle (Figures 3B,C). The patient remains asymptomatic 6 months after surgery.

## Discussion

In all published reports of aorto-ventricular tunnel, extremely rare congenital heart defect, the leaflet involved was either the right coronary leaflet, most frequently, or the left coronary leaflet, but never the anterior non-adjacent leaflet (1–25).

We are not aware of any report, an aorto-ventricular tunnel of the non-adjacent aortic leaflet in transposition of the great arteries, with, therefore, aorto-right ventricular tunnel.

The patient we report underwent arterial switch in the neonatal period, but at that time no one observed the presence of native aortic valve regurgitation in the right ventricle, while regular reports have been done on the presence of pulmonary (previous aortic) valve regurgitation from the age of 1 year.

We tried to review all investigations available from the neonatal period, and we found an echocardiography recorded before the arterial switch, showing some evidence of native aortic valve regurgitation, but the quality of the imaging was too poor for printing.

Based on this observation, and on the intra-operative findings, we can assume that our patient presented with aorto-right ventricular tunnel in transposition of the great arteries. Evidently, the degree of aortic valve regurgitation at that time was only mild and the attention has been given only to the management of transposition of the great arteries with ventricular septal defect. Over the time, the regurgitation of the new pulmonary valve progressively increased, with subsequent dilatation of the right ventricle, to the point of requiring surgery because of excessive end-diastolic and end-systolic right ventricular volume. And after having found the morphology described, the plan of implanting a pulmonary valve has been changed to the repair of the disconnected non-adjacent leaflet.

## Concluding Remarks

A very unusual combination of aorto-ventricular tunnel with transposition of the great arteries came to observation 18 years after the arterial switch performed in the neonatal period, due to progressive regurgitation of the native aortic valve in pulmonary position. Surgical repair of the lesion with attachment of the non-adjacent leaflet to the annulus avoided the planned valve implantation.

## Ethics Statement

Written and informed consent was obtained from the patient for publication of this case report.

## Author Contributions

AFC proposed the report of this malformation never observed before, prepared the entire manuscript, and the revised edition. SD contributed to the preparation of the manuscript and the choice of the illustrations. RA revised and edited the manuscript after contributing to the literature review.

## Conflict of Interest Statement

The authors declare that the research was conducted in the absence of any commercial or financial relationships that could be construed as a potential conflict of interest.

## References

[1] Corno AF Aorto-left ventricular tunnel. In: Corno AF, editor. Congenital Heart Defects. Decision Making for Surgery. Volume 2. Less Common Defects. Darmstadt: Steinkopff Verlag (2004). p. 221–8.

[2] Edwards JE Burchell HB The pathological anatomy of deficiences between the aortic root and the heart, including aortic sinus aneurysms. Thorax (1957) 12:125–39. 10.1136/thx.12.2.125 13442955 PMC1019237

[3] Levy MJ Lillehei CW Anderson RC Amplatz K Edwards JE Aortico-left ventricular tunnel. Circulation (1963) 27:841–53. 10.1161/01.CIR.27.4.841

[4] Cooley RN Harris LC Rodin AE Abnormal communication between the aorta and left ventricle: aortico-left ventricular tunnel. Circulation (1965) 31:564 10.1161/01.CIR.31.4.564 14275995

[5] Roberts WC Morrow AG Aortico-left ventricular tunnel: a cause of massive aortic regurgitation and of intracardiac aneurysm. Am J Med (1965) 39:662–7. 10.1016/0002-9343(65)90087-2 5831903

[6] Soulie P Caramanian M Pernot JM Pauly-Laubry C Left aorto-ventricular communication or tunnel. Arch Mal Coeur Vaiss (1966) 59:820–42. 4957271

[7] Bove KE Schwartz DC Aortico-left ventricular tunnel: a new concept. Am J Cardiol (1967) 19:696–709. 10.1016/0002-9149(67)90475-4 6023466

[8] Bharati S Lev M Cassels DE Aortico-right ventricular tunnel. Chest (1973) 63:198–202. 10.1378/chest.63.2.198 4688066

[9] Pérez-Martinez V Quero M Castro C Moreno F Brito JM Merino G Aortico-left ventricular tunnel: a clinical and pathologic review of this uncommon entity. Am Heart J (1973) 85:237–45. 10.1016/0002-8703(73)90465-1 4688833

[10] Saylam A Tuncali T Ikizler C Aytac A Aorto-right ventricular tunnel: a new concept in congenital cardiac malformations. Ann Thorac Surg (1974) 18:634–7. 10.1016/S0003-4975(10)64412-8 4433180

[11] Norwicki ER Aberdeen E Friedman S Rashkind WJ. Congenital left aortic sinus-left ventricle fistula and review of aortocardiac fistulas. Ann Thorac Surg (1977) 23:378–88. 10.1016/S0003-4975(10)64149-5 849055

[12] Sung CS Leachman RD Zerpa F Angelini P Lufschanowski R Aortico-left ventricular tunnel. Am Heart J (1979) 98:87–93. 10.1016/0002-8703(79)90324-7 453015

[13] Perry JC Nanda NC Kicks DG Harris JP Two-dimensional echocardiographic identification of aortico-left ventricular tunnel. Am J Cardiol (1983) 52:913–4. 10.1016/0002-9149(83)90443-5 6624687

[14] Fripp RR Werner JC Whitman V Nordenberg A Waldhausen JA. Pulsed Doppler and two-dimensional echocardiographic findings in aortico-left ventricular tunnel. J Am Coll Cardiol (1984) 4:1012. 10.1016/S0735-1097(84)80064-9 6491067

[15] Bash SE Huhta JC Nihill MR Vargo TA Hallman GL Aortico-left ventricular tunnel with ventricular septal defect: two dimensional/Doppler echocardiographic diagnosis. J Am Coll Cardiol (1985) 5:757–60. 10.1016/S0735-1097(85)80408-3 3973274

[16] Grant P Abrams LD De Giovanni JV Shah KJ Silove E Aortico-left ventricular tunnel arising from the left ventricular sinus. Am J Cardiol (1985) 55:1657–8. 10.1016/0002-9149(85)91001-X 4003319

[17] Humes RA Hagler DJ Julsrud PR Levy JM Feldt RH Schaff HV. Aortico-left ventricular tunnel: diagnosis based on two-dimensional echocardiography, color flow Doppler imaging, and magnetic resonance imaging. Mayo Clin Proc (1986) 61:901–7. 10.1016/S0025-6196(12)62613-5 3531735

[18] Tuna IC Edwards JE Aortico-left ventricular tunnel and aortic insufficiency. Ann Thorac Surg (1988) 45:5–6. 10.1016/S0003-4975(10)62383-1 3337576

[19] Kafka H Chan KL Leach AJ Asymptomatic aortico-left ventricular tunnel in adulthood. Am J Cardiol (1989) 63:1021–2. 10.1016/0002-9149(89)90168-9 2929461

[20] Kakadekar AP Sandor GGS Patterson MW LeBlanc JG. Role of transesophageal echocardiography in the management of aortic-left ventricular tunnel. Pediatr Cardiol (1995) 16:137–40. 10.1007/BF00801913 7617509

[21] Ho SY Muriago M Cook AC Thiene G Anderson RH. Surgical anatomy of aorto-left ventricular tunnel. Ann Thorac Surg (1998) 65:509–14. 10.1016/S0003-4975(97)01083-7 9485255

[22] Talwar S Choudhary UK Kothari SS Airan B Aortico-right ventricular tunnel. Int J Cardiol (1999) 31:201–5. 10.1016/S0167-5273(99)00059-5 10454311

[23] Grab D Paulus WE Terinde R Lang D. Prenatal diagnosis of an aortico-left ventricular tunnel. Ultrasound Obstet Gynecol (2000) 15:435–8. 10.1046/j.1469-0705.2000.00119.x 10976489

[24] McKay R Anderson RH Cook AC The aorto-ventricular tunnels. Cardiol Young (2002) 12:563–80. 10.1017/S1047951102001038 12636007

[25] Anderson RH Cook AC Brown NA Henderson DJ Chaudhry B Mohun T Development of the outflow tracts with reference to aortopulmonary window and aortoventricular tunnels. Cardiol Young (2010) 20(Suppl3):92–9. 10.1017/S1047951110001137 21087564

[26] Chessa M Chaudhari M De Giovanni JV Aorto-left ventricular tunnel: transcatheter closure using an Amplatzer duct occluder device. Am J Cardiol (2000) 86:253–4. 10.1016/S0002-9149(00)00873-0 10913500

[27] Bernhard WF Plauth W Fyler D Unusual abnormalities of the aortic root or valve necessitating surgical correction in early childhood. N Engl J Med (1970) 282:68–71. 10.1056/NEJM197001082820204 5409499

[28] Somerville J English T Ross DN Aorto-left ventricular tunnel: clinical features and surgical management. Br Heart J (1974) 36:321–8. 10.1136/hrt.36.4.321 4276400 PMC1020026

[29] Mair DD Fulton RE McGoon DC. Successful surgical repair of aortico-left ventricular tunnel in an infant. Mayo Clin Proc (1975) 50:691–6. 1195778

[30] Edwards JE Aortico-left ventricular tunnel: the case for early treatment. Chest (1976) 70:5–6. 10.1378/chest.70.1.5 1277932

[31] Nichols GM Lees MH Henken DP Sunderland CO Starr A. Aortico-left ventricular tunnel: recognition and repair in infancy. Chest (1976) 70:74–7. 10.1378/chest.70.1.74 1277936

[32] Okoroma EO Perry LW Scott LP McClenathan JE Aortico-left ventricular tunnel: clinical profile, diagnostic features, and surgical considerations. J Thorac Cardiovasc Surg (1976) 71:238–43. 1246149

[33] Spooner EW Dunn JM Behrendt DM Aortico-left ventricular tunnel and sinus of Valsalva aneurysm: case report with operative repair. J Thorac Cardiovasc Surg (1978) 75:232–6. 625129

[34] Björk VO Eklöf O Wallgren G Zetterqvist P Successful surgical treatment of an aortico-left ventricular tunnel in a four-month-old infant. J Thorac Cardiovasc Surg (1979) 78:35–8. 156290

[35] Villani M Tiraboschi R Marino A De Tommasi M Velitti F Giani PC Aortico-left ventricular tunnel in infancy: two surgical cases. Scand J Thorac Cardiovasc Surg (1980) 14:169–75. 10.3109/14017438009100993 7433936

[36] Ruschewski W de Vivie ER Kirchhoff PG Aortico-left ventricular tunnel. J Thorac Cardiovasc Surg (1981) 29:282 10.1055/s-2007-1023496 6179201

[37] Levy MJ Schachner A Blieden LC. Aortico-left ventricular tunnel: collective review. J Thorac Cardiovasc Surg (1982) 84:102. 7087526

[38] Turley K Silverman NH Teitel D Mavroudis C Snider R Rudolph AM. Repair of aortico-left ventricular tunnel in the neonate: surgical, anatomic and echocardiographic considerations. Circulation (1982) 65:1015–20. 10.1161/01.CIR.65.5.1015 7074737

[39] Björk VO Hongo T Aberg B Bjarke B. Surgical repair of aortico-left ventricular tunnel in a 7-day-old child. Scand J Thorac Cardiovasc Surg (1983) 17:185–9. 10.3109/14017438309099350 6359390

[40] Serino W Andrade JL Ross DN de Leval MR Somerville J. Aorto-left ventricular communication after closure: late postoperative problems. Br Heart J (1983) 49:501–6. 10.1136/hrt.49.5.501 6838737 PMC481338

[41] Ribeiro P Bun-Tan LB Oakley CM. Management of aortic left ventricular tunnel. Br Heart J (1985) 54:333–6. 10.1136/hrt.54.3.333 4041303 PMC481905

[42] Meldrum-Hanna W Schroff R Ross DN. Aortico-left ventricular tunnel: late follow-up. Ann Thorac Surg (1986) 42:304–6. 10.1016/S0003-4975(10)62740-3 3753078

[43] Deuvaert FE Goffin Y Wellens F De Paepe J Primo G Aortico-left ventricular tunnel (ALVT): a diagnostic and surgical “must”. Acta Cardiol (1986) 41:53–62. 3485872

[44] Hovaguimian H Cobanoglu A Starr A. Aortico-left ventricular tunnel: a clinical review and new surgical classification. Ann Thorac Surg (1988) 45:106–12. 10.1016/S0003-4975(10)62413-7 3276275

[45] Lindberg H Ovrum E Bjornstad PG Stake G Pedersen T. Surgical repair of aortico-left ventricular tunnel (ALVT). Scand J Thorac Cardiovasc Surg (1988) 22:285–7. 10.3109/14017438809106077 3227331

[46] Warnke H Bartel J Blumenthal-Barby C. Aortico-ventricular tunnel. Thorac Cardiovasc Surg (1988) 36:86–8. 10.1055/s-2007-1020050 3388408

[47] Akalin H Erol C Oral D Corapcioglu T Ucanok K Ozyurda U Aortico-left ventricular tunnel: successful diagnostic and surgical approach to the oldest patient in the literature. J Thorac Cardiovasc Surg (1989) 97:804–5. 2709873

[48] Hucin B Horvath P Skrovanek J Reich O Samanek M. Correction of aortico-left ventricular tunnel during the first day of life. Ann Thorac Surg (1989) 47:254–6. 10.1016/0003-4975(89)90282-8 2645839

[49] Duveau D Baron O Michaud JL Lefevre M Laboux L Dupon H Aortico-left ventricular tunnel: long-term follow-up, therapeutic implications. Arch Mal Coeur Vaiss (1989) 82:785–9. 2500103

[50] Anderson RH Surgical treatment of aortico-left ventricular tunnel. Eur J Cardiothorac Surg (1991) 5:443–4. 10.1016/1010-7940(91)90194-O 1910855

[51] Horvath P Balaji S Skovranek S Hucin B de Leval MR Stark J. Surgical treatment of aortico-left ventricular tunnel. Eur J Cardiothorac Surg (1991) 5:113–6. 10.1016/1010-7940(91)90208-2 2025436

[52] Sreeram N Franks R Arnold R Walsh K. Aortico-left ventricular tunnel: long-term outcome after surgical repair. J Am Coll Cardiol (1991) 17:950–5. 10.1016/0735-1097(91)90878-D 1999633

[53] Rosenkranz ER Murphy DJ Aortico-left ventricular tunnel in a neonate. Cleve Clin J Med (1992) 59:87–90. 10.3949/ccjm.59.1.87 1551219

[54] Zannini L Gargiulo G Albanese SB Bonvicini M Santorelli MC Frascaroli G Successful surgical repair of an aortico-left ventricular tunnel in a two-day old child. J Cardiovasc Surg (1992) 33:295–7. 1534810

[55] Rosengart TK Redel DA Stark JF. Surgical repair of aorto-right ventricular tunnel in an infant. Ann Thorac Surg (1993) 55:520–2. 10.1016/0003-4975(93)91034-K 8431072

[56] Chen YF Chiu CC Wu JR. Correction of aortico-left ventricular tunnel in a small oriental infant: a brief clinical review. J Cardiovasc Surg (1994) 35:71. 8120083

[57] Cook AC Fagg NLK Ho SY Groves AMM Sharland GK Anderson RH Echocardiographic-anatomical correlations in aorto-left ventricular tunnel. Br Heart J (1995) 74:443–8. 10.1136/hrt.74.4.443 7488462 PMC484054

[58] Sousa-Uva M Touchot A Fermont L Piot D Delezoide AL Serraf A Aortico-left ventricular tunnel in fetuses and infants. Ann Thorac Surg (1996) 61:1805–10. 10.1016/0003-4975(96)00189-0 8651788

[59] Lukacs L Richter T Kadar K. Aortico-left ventricular tunnel: late reoperations. Cardiovasc Surg (1997) 5:439–42. 10.1016/S0967-2109(97)00039-2 9350803

[60] Grünenfelder J Zünd G Prêtre R Schmidli J Vogt PR Turina MI Right coronary artery from aorto-left ventricular tunnel: case report of a new surgical approach. J Thorac Cardiovasc Surg (1998) 116:363–5. 10.1016/S0022-5223(98)70143-6 9699596

[61] Michielon G Sorbara C Casarotto DC. Repair of aortico-left ventricular tunnel originating from the left aortic sinus. Ann Thorac Surg (1998) 65:1780–3. 10.1016/S0003-4975(98)00211-2 9647106

[62] Vargas FJ Molina A Martinez JC Ranzini ME Vazquez JC. Aortico-right ventricular tunnel. Ann Thorac Surg (1998) 66:1793–5. 10.1016/S0003-4975(98)00927-8 9875792

[63] Diamant S Luber JM Gootman N. Successful repair of aortico-left ventricular tunnel associated with severe aortic stenosis in a newborn. Pediatr Cardiol (1985) 6:171–5. 10.1007/BF02336559 4080577

[64] Guyton RA Michalik RE McIntyre AB Plauth WH Nugent EW Hatcher CR Aortic atresia and aortico-left ventricular tunnel: successful surgical management by Konno aortoventriculoplasty in a neonate. J Thorac Cardiovasc Surg (1986) 92:1099–101. 3784589

[65] Knott-Craig CJ vander Merwe PL Kalis NN Hunter J. Repair of aortico-left ventricular tunnel associated with subpulmonary obstruction. Ann Thorac Surg (1992) 54:557–9. 10.1016/0003-4975(92)90455-D 1510526

[66] Bitar FF Smith FC Kavey RE Kveselis DA Byrum CJ Brandt B Aortico-left ventricular tunnel with aortic atresia in the newborn. Am Heart J (1993) 126:1480–2. 10.1016/0002-8703(93)90553-L 8249811

[67] Martin Jimenez J Gonzalez Dieguez CC Quero Jimenez C Rico Gomez F Bermudez Canete R Quero Jimenez M Aortico-left ventricular tunnel associated with pulmonary valve stenosis. Rev Esp Cardiol (1996) 49:921–4. 9026844

[68] Weldner P Dhillon R Taylor JF de Leval MR. An alternative method for repair of aortico-left ventricular tunnel associated with severe aortic stenosis presenting in a newborn. Eur J Cardiothorac Surg (1996) 10:380–2. 10.1016/S1010-7940(96)80098-4 8737696

[69] Rauzier JM Bonnet D Zniber L Sidi D Aggoun Y Acar P Aortic-ventricular tunnel with right coronary artery atresia. Arch Mal Coeur Vaiss (1997) 90:725–7. 9295958

[70] Hruda J Hazekamp MG Sobotka-Plojhar MA Ottenkamp J. Repair of aorto-right ventricular tunnel with pulmonary stenosis and an anomalous origin of the left coronary artery. Eur J Cardiothorac Surg (2002) 21:1123–5. 10.1016/S1010-7940(02)00115-X 12048098

